# International Exhibition for 1876

**Published:** 1872-12-01

**Authors:** Joseph R. Hawley, Lewis Walk Smith

**Affiliations:** President; Temporary Sec’y.


### INTERNATIONAL EXHIBITION FOR 1876.

We have received from Dr. F. L. Mathers, of Carlinville, Ill., one of the United States Centennial Commissioners for this State, the following address, and we insert it for the information of our readers, and hope they will give it due attention.—Editor.

### An Address by the United States Centennial Commission.

*To the People of the United States:*

The Congress of the United States has enacted that the completion of the One Hundredth Year of American Independence shall be celebrated by an International Exhibition of the Arts, Manufactures, and Products of the soil and mine, to be held at Philadelphia, in 1876, and has appointed a Commission, consisting of representatives from each State and Territory, to conduct the celebration.

Originating under the auspices of the National Legislature, controlled by a National Commission, and designed as it is to "Commemorate the first Century of our existence, by an Exhibition of the Natural resources of the Country and their development, and of our progress in those Arts which benefit mankind, in comparison with those of older Nations," it is to the people at large that the Commission look for the aid which is necessary to make the Centennial Celebration the grandest anniversary the world has ever seen.

That the completion of the first century of our existence should be marked by some imposing demonstration is, we believe, the patriotic wish of the people of the whole country. The Congress of the United States has wisely decided that the Birth-day of the Great Republic can be most fittingly celebrated by the universal collection and display of all the trophies of its progress. It is designed to bring together, within a building covering fifty acres, not only the varied productions of our mines and of the soil, but types of all the intellectual triumphs of our citizens, specimens of everything that America can furnish, whether from the brains or the hands of her children, and thus make evident to the world the advancement of which a self governed people is capable.

In this "Celebration" all nations will be invited to participate; its character being International. Europe will display her arts and manufactures, India her curious fabrics, while newly opened China and Japan will lay bare the treasures which for centuries their ingenious people have been perfecting. Each land will compete in generous rivalry for the palm or superior excellence.

To this grand gathering every zone will contribute its fruits and cereals. No mineral shall be wanting; for what the East lacks the West will supply. Under one roof will the South display in rich luxuriance her growing cotton, and the North in miniature, the ceaseless machinery of her mills converting that cotton into cloth. Each section of the globe will send its best offerings to this exhibition, and each State of the Union, as a member of one united body politic, will show to her sister States and to the world, how much she can add to the greatness of the nation of which she is a harmonious part.

To make the Centennial Celebration such a success as the patriotism and the pride of every American demands will require the co-operation of the people of the whole country. The United States Centennial Commission has received no Government aid, such as England extended to her World's Fair, and France to her Universal Exposition, yet the labor and responsibility imposed upon the Commission is as great as in either of those undertakings. It is estimated that ten millions of dollars will be required, and this sum Congress has provided shall be raised by stock subscription, and that the people shall have the opportunity of subscribing in proportion to the population of their respective States and Territories.

The Commission looks to the unfailing patriotism of the people of every section, to see that each contributes its share to the expenses, and receives its share of the benefits of an enterprise in which all are so deeply interested. It would further earnestly urge the formation in each State and Territory of a centennial organization, which shall in time see that county associations are formed, so that when the nations are gathered together in 1876 each Commonwealth can view with pride the contributions she has made to the national glory.

Confidently relying on the zeal and patriotism ever displayed by our people in every national undertaking, we pledge and prophecy, that the Centennial Celebration will worthily show how greatness, wealth and intelligence, can be fostered by such institutions as those which have for one hundred years blessed the people of the United States.

JOSEPH R. HAWLEY, *President.*

LEWIS WALN SMITH, *Temporary Sec'y.*

WE have received the following circular from Messrs. Codman & Shurtleff, Boston:

To remove the doubts that will naturally arise respecting our means of filling orders since the disastrous fire, but just extinguished, which has laid in ruins most of the business part of our city. We are so fortunate as to have entirely escaped all direct loss: our store, factories, stock and machinery are in their usual condition, no attempt at removal even having been necessary.

We therefore join with our patrons in the sympathy we are sure will be universally felt for those less fortunate than ourselves. We thank our friends for their liberal patronage in the past, and assure them that our facilities for meeting their wants, and our desire to do so to their entire satisfaction, remain undiminished.

CODMAN & SHURTLEFF.

